# La capacidad de resiliencia de la medicina de laboratorio durante la pandemia de la enfermedad por coronavirus (COVID-19) iniciada en 2019

**DOI:** 10.1515/almed-2020-0039

**Published:** 2020-05-19

**Authors:** Giuseppe Lippi, Mario Plebani

**Affiliations:** Sección de Bioquímica Clínica, Azienda ospedaliera universitaria integrata di Verona, Verona, Italy; Departamento de Medicina de Laboratorio, Hospital Universitario de Padua, Padua, Italy

En las últimas décadas, los tests de diagnóstico *in vitro* han sido objeto de reiterados y, a menudo irracionales, recortes, lo que ha contribuido a reducir los recursos técnicos y humanos en los laboratorios de todo el mundo [1]. Aunque, la mayoría de laboratorios están acostumbrado a trabajar bajo mínimos [2], la pandemia de enfermedad por coronavirus iniciada en 2019 (COVID-19) ha provocado una crisis mundial sin precedentes, que ha sobrepasado rápidamente la capacidad de respuesta de todo el sistema público de salud, incluida la de los laboratorios en los que se realizan las pruebas diagnósticas [3]. En su último informe, la Organización Mundial de la Salud (OMS) indica que más de 600.000 personas se han visto afectadas por el COVID-19 en todo el mundo, habiendo éste causado unas 33.000 muertes [4]. Especialmente alarmantes son las estadísticas epidemiológicas en algunos países como España e Italia donde, en poco tiempo, se alcanzará la cifra de 100.000 personas afectadas, con índices de mortalidad de entre el 8% y el 11% [4], similares a los del brote de síndrome respiratorio agudo (SARS, por sus siglas en inglés) anterior, causado por un coronavirus análogo [5]. Además, cerca del 20% de todos los casos de COVID-19 requieren hospitalización, bien en las unidades de cuidados intensivos, bien en cuidados críticos, lo que supone una carga adicional para los laboratorios clínicos, que deben proporcionar los resultados de tests críticos (esto es, urgentes) en el menor tiempo posible [6].

En esta "tormenta perfecta", una vez más, la medicina de laboratorio está demostrando su, por otro lado ya conocida, inherente resiliencia, que está permitiendo a los especialistas en medicina de laboratorio proporcionar de manera ininterrumpida los resultados de los análisis, para el diagnóstico, pronóstico y manejo de los pacientes con COVID-19. Existen varias líneas de evidencia que demuestran que el diagnóstico etiológico de la infección por SARS-CoV-2 (el coronavirus responsable del COVID-19) no sería posible sin los análisis de laboratorio, ya que estos permiten la identificación del virus en muestras biológicas mediante reacción en cadena de la polimerasa con transcriptasa inversa (RT-PCR) o a través del análisis de la respuesta inmunológica frente al virus, analizando la producción de anticuerpos. Aunque la precisión diagnóstica de la RT-PCR a partir únicamente de hisopos orofaríngeos y nasofaríngeos y de los análisis serológicos en suero o plasma no es la ideal, especialmente si estas pruebas se realizan en la misma semana en la que aparecieron los primeros síntomas (lo que se supone el 67% y el 38% de los casos, respectivamente), la combinación de estas dos técnicas diagnósticas puede mejorar la sensibilidad del diagnóstico en el laboratorio clínico en un 80% [7]. Entre 8 y 14 días tras la aparición de los síntomas, la sensibilidad combinada de la identificación de ARN del virus y la detección de anticuerpos se incrementa hasta el 97%, lo cual cumple los criterios óptimos de diagnóstico.

Además de contribuir al diagnóstico de las infecciones por SARS-CoV-2, la medicina de laboratorio es también esencial en la estratificación del riesgo, ya que los hemogramas y el análisis de biomarcadores inflamatorios, cardíacos, musculares, hepáticos, renales y de hemostasia son esenciales a la hora de identificar a aquellos pacientes con un mayor riesgo de desarrollar las complicaciones más graves del COVID-19, como el síndrome de dificultad respiratoria aguda (SDRA), el síndrome de respuesta inflamatoria sistémica (SRIS), el fallo multiorgánico, e incluso la muerte [8].

En este momento, ni los gobiernos ni las gerencias de los hospitales pueden negar que el brote de COVID-19 está causando la saturación de una sanidad pública que se encontraba ya de por sí cerca del abismo. Aunque no contamos con una bola de cristal con la que podamos predecir cuándo o cómo esta pandemia sin precedentes terminará, en caso de que lo haga, debemos extraer algunas conclusiones. La respuesta local de los servicios de medicina de laboratorio al brote de COVID-19 en todo el mundo siempre ha sido eficaz, a pesar de los reiterados recortes. Debemos dar un reconocimiento al personal de los laboratorios clínicos por la eficiencia con la que realizan su labor. No obstante, dado que hasta ahora se ha estado trabajando con los recursos mínimos para poder realizar nuestro trabajo, se ha tenido que reclutar personal de manera urgente, a menudo mediante selecciones rápidas, así mismo habiendo tenido que adquirir más instrumental para poder hacer frente al incremento en la demanda de análisis, especialmente en áreas como la de biología molecular y serología. La escasez de algunos reactivos debido a las limitaciones impuestas a la acumulación de stock para reducir el gasto sanitario o a la incapacidad de los proveedores para responder a la demanda, han empeorado la vulnerabilidad de este sector. Por último, la incesante absorción de pequeños laboratorios por otros de mayor tamaño ha contribuido a aumentar la demora en los diagnósticos, retrasando de este modo la toma de decisiones clínicas en muchos lugares del mundo [9].

De este modo, concluimos citando el famoso dicho en latín “*herrare humanum est, perseverare autem diabolicum*”, con el más sincero deseo de que seamos capaces de extraer un aprendizaje de la tragedia que estamos viviendo.
